# Current driven transition from Abrikosov-Josephson to Josephson-like vortex in mesoscopic lateral S/S’/S superconducting weak links

**DOI:** 10.1038/srep35694

**Published:** 2016-10-18

**Authors:** G. Carapella, P. Sabatino, C. Barone, S. Pagano, M. Gombos

**Affiliations:** 1 Dipartimento di Fisica E.R. Caianiello and CNR-SPIN UOS Salerno, Università di Salerno, I-84084 Fisciano, Salerno, Italy; 2 CNR-IMM UOS Napoli, Italy

## Abstract

Vortices are topological defects accounting for many important effects in superconductivity, superfluidity, and magnetism. Here we address the stability of a small number of such excitations driven by strong external forces. We focus on Abrikosov-Josephson vortex that appears in lateral superconducting S/S’/S weak links with suppressed superconductivity in S’. In such a system the vortex is nucleated and confined in the narrow S’ region by means of a small magnetic field and moves under the effect of a force proportional to an applied electrical current with a velocity proportional to the measured voltage. Our numerical simulations show that when a slow moving Abrikosov-Josephson vortex is driven by a strong constant current it becomes unstable with respect to a faster moving excitation: the Josephon-like vortex. Such a current-driven transition explains the structured dissipative branches that we observe in the voltage-current curve of the weak link. When vortex matter is strongly confined phenomena as magnetoresistance oscillations and reentrance of superconductivity can possibly occur. We experimentally observe these phenomena in our weak links.

In the last decade superconductivity at reduced dimensionality has received a growing interest mainly due to the huge advancements in nano-fabrication techniques that have permitted exploration and discovery of new physical phenomena when approaching the mesoscopic limit. Thin films of type II superconductors are in the mesoscopic limit when their thickness is much smaller than the London penetration depth *λ*^1^, and have at least a lateral dimension intermediate between the Ginzburg-Landau coherence length *ξ* and the Pearl length Λ^1^. Effects due to strong confinement of vortex matter^2^ in mesoscopic superconducting films, such as critical current and magnetoresistance oscillations^3^,^4^,^5^, counterintuitive reentrance of superconductivity caused by magnetic field^6^,^7^ or realization of a Weber blockade in a superconducting material^8^, have been recently demonstrated. The mechanism of phase slippage^1^,^9^,^10^ and related concept of kinematic vortex^11^,^12^, previously introduced to account for transport properties of micro-bridges and whiskers, has gained a renewed interest also in the explanation of transport properties of plain thin superconducting films at mesoscale^13^,^14^,^15^,^16^,^17^,^18^ (with or without holes) and nanowires^19^,^20^,^21^,^22^,^23^. The same mechanism could play an important role in the dynamics of iron-based superconducting grain boundary junctions, where the presence of weak links has been identified through noise spectroscopy^24^. Moreover, nano-fabrication techniques also permitted to revisit the physics of proximity effect^25^ between a superconducting material and a non superconducting material and related Josephson effects^26^ in lateral weak links^27^ at mesoscale. Using this kind of weak links, triplet superconductivity^28^ induced in a ferromagnetic half metal^29^, weak superconductivity induced in graphene^30^, evidence for nonlocal electrodynamics in Josephson junctions and related concept of Abrikosov-Josephson vortex^31^,^32^,^33^,^34^, and direct observation^35^ of Josephson vortex cores have been reported.

Very recently, exploiting the phenomenon of noticeable reduction^36^,^37^ of superconductivity at interface between a ferromagnet and a superconducting material, mesoscopic lateral S/S’/S weak links in which the weak S’ region is made of same material as the S banks, though with reduced critical temperature, have been reported. In such kind of structure Josephson coupling^38^,^39^,^40^, also controllable by current injection^40^ from the ferromagnetic layer, has been demonstrated at temperatures near the critical temperature of banks while at lower temperatures a more complex behaviour has been found. In the present report we further address such type of lateral S/S’/S weak link, both experimentally and numerically, with particular emphasis on transport properties and vortex dynamics in the presence of magnetic field. The S’ region is achieved crossing a thin, narrow and long ferromagnetic Permalloy strip directly on the top of a thin superconducting Niobium strip in the mesoscopic regime. In the absence of magnetic field, the weak link exhibits a single phase slip line branch with appreciably large current range extension. In the presence of magnetic field, the recently predicted^41^ magnetoresistance oscillations accounted for strongly confined vortex matter in a weak superconductivity region are experimentally confirmed. Moreover, the S/S’/S weak link exhibits voltage-current curves with structured dissipative branches. The numerical analysis, performed in the framework of time dependent Ginzburg-Landau model for mesoscopic type II superconductors with inhomogeneous critical temperature, suggests that these peculiar branches are accounted for a dynamical transition from a single row of slow moving moderately anisotropic vortices (Abrikosov-Josephson vortices^31^,^32^,^33^) to a single row of faster moving definitely anisotropic vortices (Josephson-like vortices). The first experimental evidence for voltage-current curves accounted for such a current driven transition in a planar weak link, and the observation of magneto-resistance oscillations recently predicted^41^ for such type of weak links are the main results of the present report.

## Results

**Figure 1:**
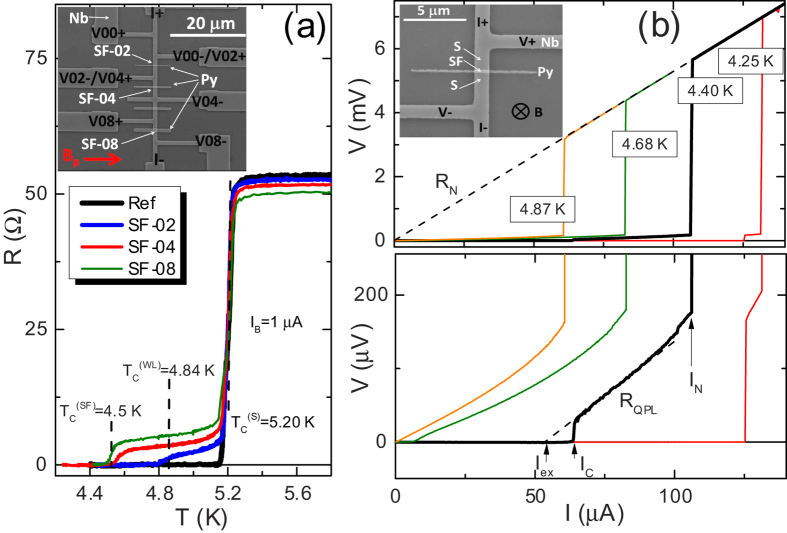
Electrical characterization of samples at zero applied magnetic field. (**a**) *R*(*T*) curves of the reference Nb strip (black line) and the Nb/NbPy/Nb lateral weak links with width of the Py strip of 0.2 *μ*m (blue line), 0.4 *μ*m (red line), and 0.8 *μ*m (green line). In the inset there is shown a micrograph of the sample layout. (**b**) *V*(*I*) curves of the weak link with 0.2 *μ*m wide Py strip recorded at several temperatures in the absence of magnetic field are shown in the top panel in full voltage range. The inset shows the micrograph of the addressed weak link. The low voltage region of the *V*(*I*) curves is shown amplified in the bottom panel.

The samples were fabricated using Niobium (Nb) as superconductor and Permalloy (Py: Ni_80_Fe_20_) as ferromagnet as described in Methods. A micrograph of final sample layout is shown in the inset of Fig. 1(a). The weak links (labeled as SF in the micrograph) are formed where the horizontal Py stripes crosses the vertical Nb strip. In this crossing region we are concerned with a Superconductor-Ferromagnet bilayer (SF) and, due to proximity effect^25^,^36^,^37^, the Nb below Py is an S’ region with superconductivity weakened with respect to the superconductivity of the uncovered S region, so that a lateral S/S’/S weak link is realized.

In the main panel of Fig. 1(a) there are shown the *R*(*T*) (resistance versus temperature) curves of the weak links with lengths 0.2 *μ*m, 0.4 *μ*m, and 0.8 *μ*m, together with the *R*(*T*) curve of reference strip. The *R*(*T*) of the 0.6 *μ*m wide weak link (not shown here to not make further heavy the plot) was found to consistently fall between the *R*(*T*) curves of the 0.4 *μ*m and the 0.8 *μ*m wide weak links. To acquire the *R*(*T*), a dc bias current *I*_*B*_ = 1 *μ*A was used, but similar results were obtained using an ac current bias or a lower dc current bias. The critical temperature of all the S banks is found to be about 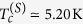, the same as the reference strip, while the critical temperature of the weak region (SF bilayer) decreases as the length of the weak link is increased and tends to saturate at the longest weak link. This is due to lateral superconducting proximity effect, i.e., the one induced by banks, that becomes negligible when separation between banks is much larger than superconducting coherence length. Assuming the lateral proximity effect as negligible already for the 0.8 *μ*m long weak link, we estimate the proximity-effect-free critical temperature (common to all weak links) of our Nb(30 nm)/Py(30 nm) bilayer as approximately 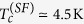. In the following we will concentrate on the 0.2 *μ*m long weak link, where, instead, a relevant lateral proximity effect can be inferred to exist for a quite large temperature window. From the *R*(*T*) curve shown in Fig. 1(a) we estimate a critical temperature 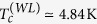 for this laterally proximized weak link. Thus, we can conclude that it represents a S/S’/S weak link in the temperature window 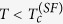, a proximized S/N/S (existence of a measurable critical current) in the temperature range 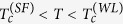, and an ordinary S/N/S structure (unmeasurably small critical current) for 
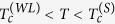
.

We notice that in Fig. 1(a) the fully normal state resistance a 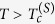 is not exactly the same for all samples. As explained in Methods, this is it not due to a non uniform Nb or lithography errors, but rather to the presence of a Nb/Py bilayer with variable length between the voltage probes at fixed distance.

A zoomed micrograph of the further addressed weak link is shown in the inset of Fig. 1(b). In the top panel we show the *V*(*I*) curves at full scale of voltage recorded at several temperatures and in the absence of magnetic field. The curves are always acquired increasing the current from zero to a suitably large target value, without making a full current loop. This is because in this work we were not interested in hysteresis effects. However, the hysteresis was always found to be negligible. As can be appreciated, beyond an upper temperature-dependent critical current *I*_*N*_ all curves fall on the same fully resistive branch passing trough the origin with slope 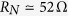, comparable to the resistance of the Nb strip in the fully normal state (but for the correction discussed in Methods). We ascribe such a branch to the transition of Nb banks to the fully normal state. For currents below *I*_*N*_ the *V*(*I*) curves are more structured, as it better evident in the bottom panel of Fig. 1(b), where the low voltage region is amplified. By increasing current from zero, a zero voltage state is observed up a temperature-dependent critical current *I*_*c*_, defined as the current at which a transition *V* = 0 → *V* ≠ 0 take place using a 0.2 *μ*V criterion, above which an intermediate dissipative branch is followed up to *I*_*N*_, as shown in Fig. 1(b). Approximately the dynamical resistance *R*_*QPL*_ of this branch is comparable to the temperature-dependent resistance of the weak link region in the normal state, 2 Ω < *R*_*QPL*_ < 3 Ω, as can be appreciated from the *R*(*T*) curve reported in Fig. 1(a). Though these branches are almost linear for currents not too large with respect to *I*_*c*_ their extrapolation to the zero voltage axis points to a finite current “excess” current^27^ *I*_*ex*_, strictly reminding the branches observed^1^ in other types of weak links or in mesoscopic strips at very large driving currents and accounted for phase slip lines/kinematic vortices^11^,^12^. As will be discussed below also in our case these branches can be accounted for phase slip lines. Compared to other cases, here we observe only a single phase slip branch, due to the short length of the weak region (0.2 *μ*m). For what said above about the different regimes, the curves *V*(*I*) recorded at 4.25 K, and 4.40 K are for the weak link in the S/S’/S regime, the curve at 4.68 K is for the laterally proximized S/N/S regime, while the curve at 4.87 K belongs to the pure S/N/S regime. Below we further address the weak link at *T* = 4.25 K, i.e., the S/S’/S regime.

### Transition from Abrikosov-Josephson to Josephson-like vortex motion, and magnetoresistance oscillations

**Figure 2:**
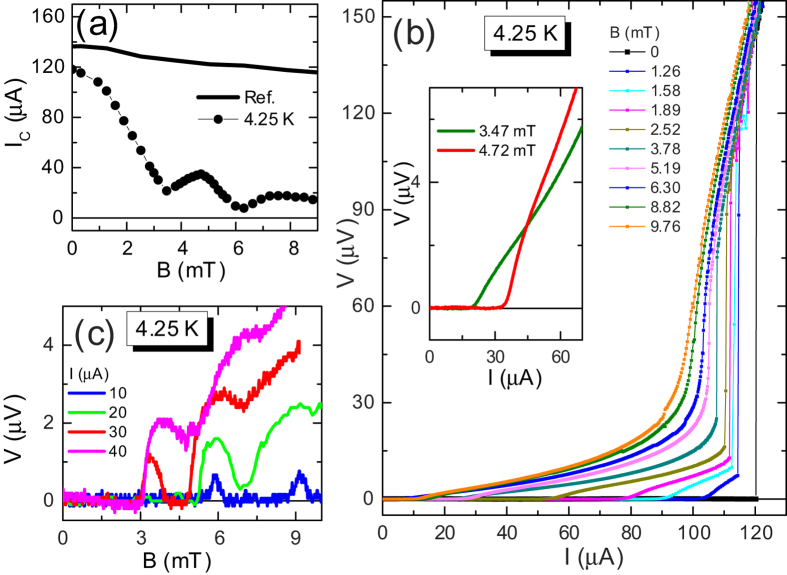
Evidence for magnetoresistance oscillations and Josephson-like vortex in action. (**a**) Modulation of critical current as a function of magnetic field, *I*_*c*_(*B*), recorded at *T* = 4.25 K (solid circles). The *I*_*c*_(*B*) of the reference sample (solid line) is also shown for comparison. (**b**) *V*(*I*) curves recorded for several values of applied magnetic field. The inset shows a magnification of the curves recorded at magnetic fields corresponding to the first minimum and first relative maximum of the *I*_*c*_(*B*) pattern shown in panel (a). (**c**) Voltage versus magnetic field, *V*(*B*), curves recorded for several values of fixed bias current *I*.

In S/S’/S weak links with the length of S’ region suitably small (not too large with respect to coherence length at working temperature) a modulation of the critical current as a function of magnetic field could be expected, due to the presence of a small number of strongly confined vortices^4^,^5^,^41^,^42^ or, more simply, to the presence of a Josephson coupling^26^. In our sample we observe critical current oscillations. The modulation of critical current as a function of magnetic field, *I*_*c*_(*B*), recorded at *T* = 4.25 K, is shown in Fig. 2(a). The magnetic field is applied perpendicular to the substrate, as shown in the top inset of Fig. 1(b). We also show the *I*_*c*_(*B*) of the reference sample (solid line) for comparison. As can be appreciated, for the very small range of magnetic fields involved the critical current of the reference sample barely decreases of some percents, so we are confident that vortices in the banks are again not present^43^ for these fields and the oscillations are due only to the weak region.

For this temperature the periodicity of oscillations is not well defined, and the pattern reminds the one reported^40^ in a lateral weak link very similar to the one addressed here. More generally the pattern reminds more the one exhibited by Josephson tunnel junctions of intermediate width^26^ with overlap geometry^26^ than the one (Fraunhofer-like) exhibited by junctions of short width^26^. As explained in Methods, and confirmed in the numerical simulations, in fact this is the case for our weak link.

In Fig. 2(b) we show the *V*(*I*) curves in the low voltage region for values of applied magnetic field ranging from *B* = 0 to *B* = 9.76 mT. We checked that no asymmetry or magnetic field-induced hysteresis^44^,^45^,^46^,^47^,^48^ was present in the *V*(*I*) curves when magnetic field direction was reversed or a full magnetic loop was performed. This reassures us that the field sensed by the weak link is really homogeneous, the Py rests in a single domain state, and the thickness profile of thin Nb film is flat. At very low fields, above the field-dependent critical current *I*_*c*_(*B*), an almost linear dissipative branch with low dynamical resistance is followed. Then, after a very small non linear branch, an abrupt transition to the phase slip line branch is achieved at some critical current. By increasing the magnetic field the current range of the low resistivity branch extends, and, above a certain magnetic field value, continuously merges in the phase slip line branch. The continuous transition region exhibits a dynamical resistance larger than the *R*_*QPL*_.

The low resistivity portion of curves reminds the flux flow branches reported^49^ in mesoscopic superconducting strips subjected to a perpendicular magnetic field or the branches predicted for weak links with very high critical current density^31^,^32^,^33^. However, here also a transition, abrupt at low field and continuous at moderate fields, to a phase slip line branch is observed. As will be better clear in the next section, features of *V*(*I*) curves reported in Fig. 2(b) can be explained with presence of Abrikosov-Josephson^31^,^32^,^33^ vortex flow at low current that transforms in Josephson-like vortex flow at larger currents.

Inspection of very low voltage region suggests that to the modulation of critical current corresponds also a modulation of the dynamical resistance of the linear flux flow regime. This can be better appreciated in the inset of Fig. 2(b), where we show a magnification of the curves recorded at magnetic fields corresponding to the first minimum and first relative maximum of the *I*_*c*_(*B*) pattern at *T* = 4.25 K shown in Fig. 2(a). The resistivity of the flux flow branch at first minimum (green line) is lower than the resistivity of the branch at first maximum (red line) suggesting that mobility of vortices can be tuned^41^ by magnetic field and can possibly exhibit oscillations. In particular, as we could infer looking at the curves in the inset, if the bias current is fixed between the value of critical current at first minimum 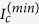 and the value of critical current at first maximum 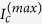, vortices in motion (meaning *V* ≠ 0) at lower field can be stopped at larger field (meaning *V* = 0), i.e., it is possible a reentrance of superconducting state when magnetic field is increased. The magneto-resistance oscillations observed in our sample are shown in Fig. 2(c), where we report voltage versus magnetic field, *V*(*B*), curves for several values of fixed bias current *I*. In particular the red curve exhibiting a reentrance of superconductivity corresponds to 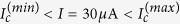.

## Discussion

In order to investigate the response of the weak link to driving current and magnetic fields, we use the time-dependent Ginzburg-Landau (TDGL) theory where the presence of weak region is included through a spatially dependent critical temperature *T*_*c*_. This approach, already used in the past^11^,^27^,^41^ to describe superconducting weak links, is based on the anisotropic Ginzburg-Landau formalism, where the weak link is described by an anisotropic expansion coefficient^1^,^25^ *α*(*T*) of the Gibbs free energy density. We use the model in its 2D simplified form. The simplification is justified when the strip exhibits a large Ginzburg-Landau parameter *κ* (definitely type-II superconductor) and it is in the mesoscopic limit^4^,^43^, i.e., it is very thin with respect to the London penetration depth (*t*_*ff*_  ≪  *λ*), sufficiently narrow with respect to the Pearl length (*W* ≤ Λ = 2*λ*^2^/*d*), and quite wider than the Ginzburg-Landau coherence length (*W*_*f*_  ≫  *ξ*). In the mesoscopic limit current density is uniform with good approximation^43^,^50^ (at least in regions far from sharp turns or geometrical non-uniformities^43^,^50^,^51^,^52^, as it is our case) and internal magnetic field corrections due to current as well as the demagnetizing effects can be usually neglected^4^,^43^,^50^.

**Figure 3:**
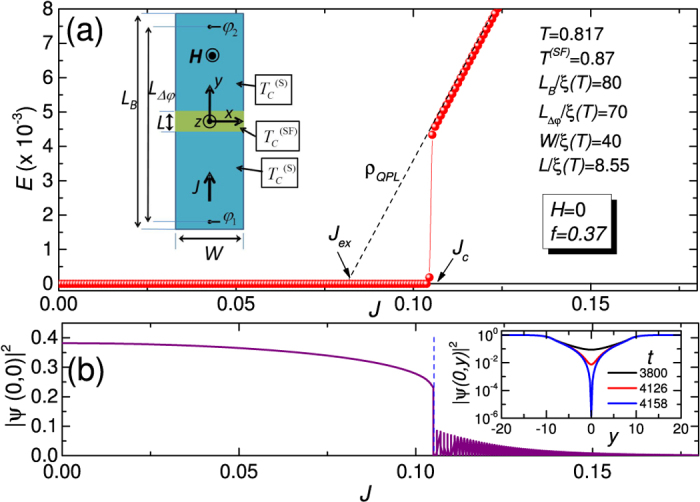
Calculated *E*(*J*) curve at *H* = 0: phase slip line branch. (**a**) Calculated time averaged electric field versus applied current density, *E*(*J*), of the weak link at zero applied magnetic field. In the inset there is shown the geometry used to model the real S/SF/S lateral weak link structure. The used reference frame and parameters of the simulations are also shown. (**b**) Squared order parameter at the center of weak link versus applied current density at zero applied magnetic field. Above the critical current density *J*_*c*_ the order parameter is oscillating with time. In the inset there is shown the profile of the squared order parameter along *y*-direction [see inset of panel (a)] at several times when the weak link is polarized on the dissipative branch.

The 2D TDGL equation for the complex order parameter *ψ* = |*ψ*|*e*^*iθ*^ in the case of our inhomogeneous superconducting strip [see inset of Fig. 3(a)] takes the form^11^,^27^,^41^


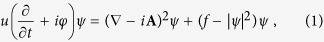


where **A** is the vector potential associated to the external magnetic field **H**, *ϕ* the electrostatic potential, *u* = 5.79 governs the relaxation of the order parameter^10^, and *f* is the anisotropy parameter discussed below. This equation is coupled with the equation for the electrostatic potential


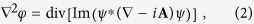


that stems from the current conservation equation **J** = **J**_*s*_ + **J**_*n*_ = [Im(ψ*(∇ − *i***A**)*ψ*)] − ∇*ϕ*. All physical quantities are measured with respect the temperature dependent critical values of the banks, as explained in Methods. The field **H** is applied in the *z*-direction and is described by **A** = (−*Hy*/2, *Hx*/2, 0). In this implicit-temperature formulation the anisotropy parameter^11^,^27^,^41^ becomes *f*(*y*) = 1 in the banks and *f*(*y*, *T*) = (*T*^(*SF*)^ − *T*)/(1 − *T*) in the weak link, where 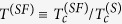. Further details on numerical integration of equations (1), and (2) are given in the Methods.

Before we proceed, we would briefly comment that the simplified 2D model (1), and (2) should be sufficient to describe the experiment. For the used dimensions, the as grown Py stripes are expected to be all in the remanent single-domain magnetic state, due to small thickness, large length-to-width aspect ratio, and sub-micrometer scale widths. However, prior the measurements we applied a magnetic field *B*_*P*_ ≈ 200 mT in the long direction of the stripes [see micrograph in Fig. 1(a)] at room temperature. This ensures that remanent single-domain magnetization is directed along the length of the strips. Moreover, in magneto-transport measurements we applied a magnetic field perpendicular to the Py strip plane up to 10 mT. We checked, using the 3D simulation package Object Oriented Micromagnetic Framework (OOMMF), that this field strength is too small to rotate the magnetization to the out-of-plane direction and that, more generally, the out-of-plane stray magnetic field component in the weak link regions (i.e., around the middle of the strip) can be neglected with respect to the applied magnetic field. This allows us to exclude any other mechanism^44^,^45^,^53^,^54^ of modulation of superconductivity beside the proximity effect at Superconductor/Ferromagnet interface. Proximity effect^1^,^25^ between a superconducting (S) material and a non superconducting material (N) manifests itself as gain of weak superconductivity in the N at expense of reduction of superconductivity in the S near the S/N interface. The reduction of superconductivity in S can be quite strong^36^,^37^ when the N material is also ferromagnetic (F). This is why we preferred to use a ferromagnetic metal to locally depress superconductivity in Nb instead of an ordinary normal metal. In our system the reduction of superconductivity in the Nb is achieved in the vertical direction of the structure [*z*-direction in the model shown in Fig. 3(a)] and accounts for the reduced critical temperature of Nb below the Py stripes. The reduction extends for a distance of the order of the coherence length *ξ*(*T*) from the S/F interface^36^,^37^, so that Nb below Py could exhibits a modulation of superconductivity along the vertical direction in our system and a full 3D model should be used. To avoid this, a thickness *t*_*f*_ lower than approximately √2 *ξ*(*T*)^1^,^25^ for the superconductor should be chosen. This is why we chose a 30 nm-thick Nb. In fact, we estimate for our Nb (see Methods) 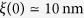, resulting in *t*_*f*_ ≤ √2 *ξ*(*T*) in the temperature range of our interest (*T *≥* *0.8). So, we can reasonably assume the superconductivity in the Nb as homogeneously depressed in the vertical direction, as also suggested by resistance measurements and magnetic pattern analysis given in Methods, and we use the simplified 2D formulation given above.

As in the experiments, we focused simulations on the shorter weak link in the S/S’/S regime achieved at *T* = 4.25 K. We chose the parameters as follows. The normalized temperature parameter that describes the weak region is extracted from the measured critical temperature of the longest weak link 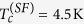 and the critical temperature of the banks 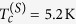 (see Fig. 1) as 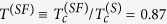. The working temperature of 4.25 K corresponds to *T* = 0.817. For this temperature the anisotropy parameter is *f*(0.817) = 0.37, while the normalized length of the weak link is *L*/*ξ*(0.817) = 8.55. The width is *W*/*ξ*(0.817) = 42.7. Having checked that value for this dimension was not particularly relevant, we chose *W*/*ξ*(0.817) = 40 in the simulations. For other two relevant lengths [see inset of Fig. 3(a)] we chose *L*_*B*_/*ξ*(0.817) = 80 and *L*_Δ*φ*_/*ξ*(0.817) = 70. When calculating time averaged electric field *E* = Δ*φ*/*L*_Δ*φ*_ to build up the *E*(*J*) curves, we take the electrostatic potential difference Δ*φ* inside the superconducting sample, a distance 5 *ξ*(*T*) away from the current injection boundaries at *y* = ± *L*_*B*_/2 = ± 40 *ξ*(*T*), as shown in Fig. 3(a). This corresponds to the four-probe configuration used in the experiment. Moreover, both current injection boundaries and voltage probes are well far from the S/S’ boundaries at *y* = ± *L*/2 = 4.275 *ξ*(*T*) of weak link region, as in the experiments.

In the main panel of Fig. 3(a) we show the calculated time averaged electric field versus applied current, *E*(*J*), curve for *H* = 0. All relevant features of experimental curve at 4.25 K [see Fig. 1(b)] are reproduced, included the dissipative branch with resistivity *ρ*_*QPL*_. To better understand such branch, in Fig. 3(b) we show the |*ψ*|^2^ at the center of weak link versus applied current density *J*. By increasing current from zero, the order parameter decreases up to a certain critical current density *J*_*c*_. Above the critical current density *J*_*c*_ the order parameter is oscillating with time, and the dissipative branch shown in Fig. 3(a) is accessed. These oscillations of order parameter can be better appreciated in the inset of Fig. 3(b), where we show the profile of the squared order parameter along *y*-direction [reference frame is shown in the inset of panel (a)] at several times when the weak link is polarized on the dissipative branch of *E*(*J*) curve. The |*ψ*|^2^ pulsates with time and periodically vanishes at the center line of the weak link. The frequency of oscillation is found to increase as the current *J* is increased. These features are typical of the celebrated phase slip line solution^9^,^10^,^11^. The only difference with seminal previous reported simulations is that here the |*ψ*|^2^ pulsates between zero and a value that is depressed with respect to the one in the banks, or, in other words, here the periodic phase slippage is localized in a region of weakened superconductivity.

**Figure 4:**
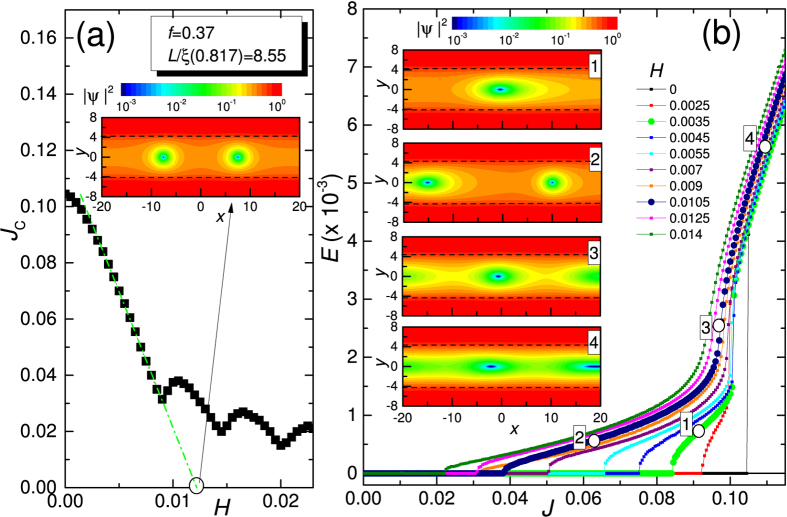
Abrikosov-Josephson vortex matter to Josephson-like vortex matter transition. (**a**) Critical current of the weak link as a function of magnetic field, *J*_*c*_(*H*). The inset is a snapshot of the squared order parameter, |*ψ*|^2^, at the marked point of *J*_*c*_(*H*). Only a portion of full simulating region is shown. The dashed lines indicates the S/S’ boundaries of the weak link region. (**b**) *E*(*J*) curves for several values of the magnetic field *H*. The numbered panels are snapshots of |*ψ*|^2^ at the marked points of the *E*(*J*) curves. Vortex matter moves from left to right.

In Fig. 4(a) we show the calculated *J*_*c*_(*H*), to be compared with the experimental *I*_*c*_(*B*) shown in Fig. 2(a): the observed oscillations of critical current are qualitatively reproduced by the simple model of equations (1) and (2). The inset in Fig. 4(a) is a snapshots of |*ψ*|^2^ at the marked point of the *J*_*c*_(*H*) curve. Here and in the following the snapshots are zooms of the full simulation region shown in Fig. 3(a), and show only parts of the fully superconducting S regions (banks) around the S’ region (weak link). The dashed lines mark the S/S’ boundaries of the weak link. The chosen point of the *J*_*c*_(*H*) curve corresponds to the critical *entry* field^4^,^43^,^49^ *H*_*s*_ and is calculated setting *J* = 0, and gradually increasing the field until at *H* = *H*_*s*_ vortex matter enters the weak link. At chosen temperature two vortices nucleate to the edges, enter the weak link and rest in the central region. As can be inferred from the snapshot relative to static vortex matter, due to the confinement in the vertical direction, in our weak link only motion of a single vortex or a single vortex row can be established by a transport current *J* > *J*_*c*_(*H*).

Finally, we should remark that a similar *J*_*c*_(*H*) and vortex matter was reported in ref. 42 for a diffusive S/N/S junction with normalized dimensions comparable to our S/S’/S weak link, calculated in the framework of Usadel model. This suggests that an S/S’/S junction qualitatively resembles a diffusive S/N/S junction.

In Fig. 4(b) there are shown the calculated *E*(*J*) curves for several values of the magnetic field *H*. These curves should be compared with the experimental data reported in Fig. 2(b). As in the experiment, there are present the low resistivity almost linear dissipative branches followed by a transition, abrupt at low field and continuous at moderate fields, to a phase slip line branch. However, when looking at the voltage jumps present in the theoretical curves of Fig. 4(b), they are less severe here than in the experiments. This may be an indication that thermal effects play some role in the experiments and cannot be captured by our (isothermal) TDGL. For example, in the experiment we cannot claim with full confidence that the high voltage regime always corresponds to a genuine phase slippage since thermal effects can simply lead to a hot spot formation. As can be appreciated below, our simplified model *qualitatively* captures the relevant experimental features but thermal effects are neglected and this possibly limits some of our claims when direct comparison with experiment is made. The numbered panels in Fig. 4(b) are snapshots of |*ψ*|^2^ at the marked points of the *E*(*J*) curves. Vortex matter moves from left to right. As evidenced by snapshots (1) and (2) the low resistivity almost linear branches are accounted for moderately anisotropic vortex flow. Snapshots (2), (3), and (4) are relative at a field *H* where by increasing the current density the *E*(*J*) curve exhibits a continuous transition of the flux flow branch to the phase slip line branch. At low current [snapshot (2)] there is a single row of two anisotropic vortices moving from left to the right. As the current is increased the transition region is accessed [snapshot (3)]. Here the vortices in the row become more anisotropic and a river of depressed superconductivity starts to form. The river of depressed superconductivity is completely formed and strongly anisotropic vortices are surfing on it when the phase slip line branch is fully accessed by a further increase of the bias current.

**Figure 5:**
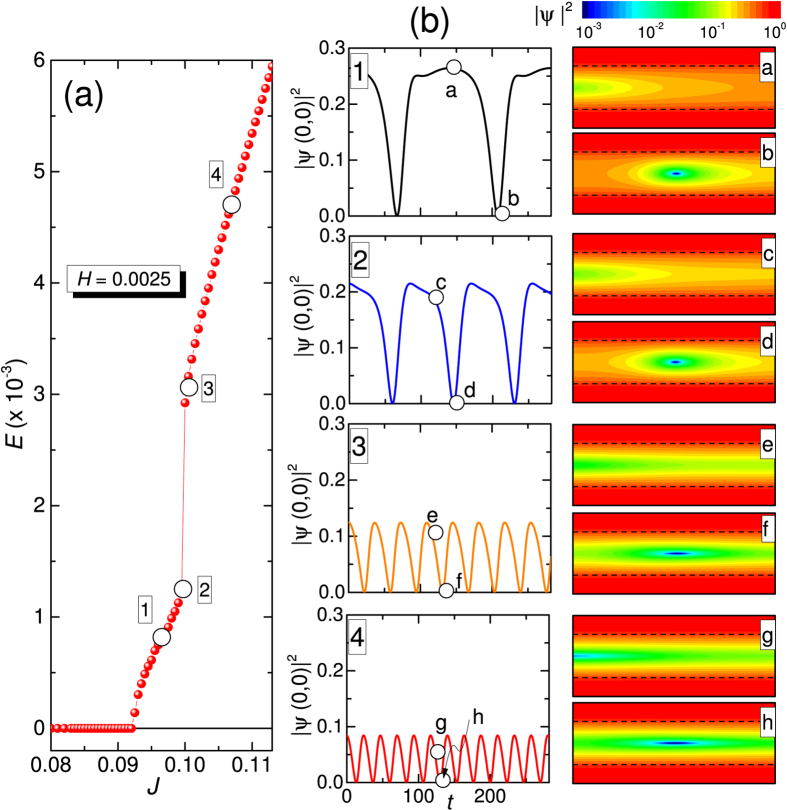
Josephson-like vortex is faster than Abrikosov-Josephson vortex. (**a**) Calculated *E*(*J*) curves at *H* = 0.0025. (**b**) The numbered panels to the left show the oscillations of the squared order parameter at center of weak link versus time when we are on the marked points of the *E*(*J*) curve shown in (**b**). The lettered panels to the right are snapshots of |*ψ*|^2^ at times marked in the left panels.

The unidirectional vortex matter motion induced by magnetic field and large transport current shown in snapshot (4) is similar to the one first reported by Vodolazov and Peeters^13^ relatively to rearrangement of the vortex lattice due to instabilities of vortex flow. In that contest, the relevant dissipative branch was named “quasi-phase-slip line” and vortex matter involved was indirectly related to the vortex-based description of a phase slip line given by Andronov *et al*.^11^. Subsequently, other groups^12^,^14^,^15^,^41^,^49^ called the fast vortex matter involved in the high resistivity branch of thin plain mesoscopic strips “kinematic vortex”. Here, instead of a plain strip we have a weak link and, to avoid confusion, we prefer to use a nomenclature typical of weak links for the vortex matter involved in the dissipative branches. Our low resistivity branch and the related vortex matter resembles the one reported^31^,^32^,^33^ for strongly overdamped Josephson junctions in the regime of nonlocal electrodynamics achieved at very large critical current of the weak link, of the order of magnitude of the depairing current of the banks. Our weak link in the S/S’/S regime falls in that regime. In a seminal work^33^ Gurevich named the anisotropic vortex matter involved in such a regime “Abrikosov-Josephson vortex”. We adopt same terminology. In agreement with finding of Gurevich^31^,^32^,^33^, the Abrikosov-Josephson vortex expands its width as the current is increased (becomes more anisotropic) resulting in a *E*(*J*) linear at low currents but more and more nonlinear, with upward curvature, when current approaches the critical current of the weak link (our local depairing current). These peculiarity can be appreciated in Fig. 4(b). We should notice that description of Abrikosov-Josephson vortex dynamics was not based on full time-dependent Ginzburg Landau model and the order parameter was assumed to be independent of transport current. This is almost the case for our low resistivity dissipative branch, before the phase slip line branch is accessed at larger currents with associated appreciable depression of order parameter (see Fig. 3). Thus, the Abrikosov-Josephson vortex matter in snapshots (1), (2), and (3) evolves in a different vortex matter shown in snapshot (4) when the high resistivity branch is accessed. Noticing that at relatively large current the order parameter becomes dynamically strongly suppressed in S’ region [see Figs 3(b) and 5)], we can roughly assume that the S/S’/S system transits to a proximized S/N/S like system and consequently the moderately anisotropic Abrikosov-Josephson vortex transforms in a definitely anisotropic vortex that we will name “Josephson-like” vortex. As will be more clear below, this current-induced transition results in a higher speed of the vortex matter and fast increase of the voltage at some current.

So that, data in Fig. 2(b) can represent an indirect experimental evidence for Abrikosov-Josephson^31^,^32^,^33^ vortex flow at low bias current that transforms in Josephson-like vortex flow at larger bias currents.

We would notice that the shape of *E*(*J*) curves in Fig. 4(b) strongly reminds the one reported in our recent work^49^ on a plain mesoscopic strip in moderate magnetic fields driven by uniform transport current. Though there are similarities in the vortex dynamics, there are some differences due to the fact that here we are concerned with a single row of vortex excitations confined in a well defined channel of depressed superconductivity. For example, in the mesoscopic strip the nonlinear flux flow region just before the instability point was accounted for by a moving glassy lattice of isotropic vortex excitations (Abrikosov) while in the present weak link it is accounted for by more and more anisotropic (as the current is increased) excitations (Abrikosov-Josephson) moving in a single row. The high resistivity branch just after the instability point in the mesoscopic strip was accounted for by a rearrangement of the vortex lattice in a channel-like structure of slightly anisotropic excitations (kinematic vortices) surfing on rivers of depressed superconductivity not existent before. Here a single river of depressed superconductivity (S’ weak link region) is present also in the absence of magnetic field and transport current and, as explained above, is further suppressed when current is large enough so that the S/S’/S system effectively transits to a proximized S/N/S (Josephson like) system and consequently the moderately anisotropic vortex transforms in a definitely anisotropic vortex that we have named “Josephson-like” vortex.

To better explain the dynamical evolution of the Abrikosov-Josephson to the faster moving Josephson-like vortex involved in our S/S’/S weak link, in Fig. 5(a) we show the *E*(*J*) at the small field *H* = 0.0025, where both almost linear flux flow branch and phase slip line branch are present. In Fig. 5(b) the numbered panels to the left show the oscillations of the squared order parameter at center of weak link versus time when we are on the marked points of the *E*(*J*) curve shown in panel (a), while the lettered panels to the right are snapshots of |*ψ*|^2^ at times marked in the left panels. As can be seen, the almost linear branch at low currents [points (1) and (2)] is accounted for a single Abrikosov-Josephson vortex moving from left to the right. The |*ψ*|^2^(*t*) vanishes each time the vortex core passes for the center of the weak link and restores when vortex core is near one of the edges. Being involved only one vortex excitation, the oscillation frequency of the |*ψ*|^2^(*t*) is a measure of the mean velocity of the vortex, as it is also the measured *E*. From this we deduce that vortex velocity is almost linearly increased with current *J* from bias point (1) to bias point (2), similar to the ordinary vortex flow. However, when with a very small increment of current the phase slip branch is accessed at bias point (3) the mean velocity abruptly increases of about a factor three and further increases as the current is further increased at bias point (4). So, on the phase slip line branch the vortex matter (Josephson-like vortex) move faster than the Abrikosov-Josephson vortex involved in the almost linear lower resistivity flux flow branch. The dynamic Abrikosov-Josephson vortex involved in the flux flow branch is elongated along the direction of motion in our weak link, and its width increases with current^31^,^32^,^33^. However, the vortex core preserve a definite spatial extension, as can be appreciated from impulsive character of |*ψ*|^2^(*t*) waveform and the relevant stroboscopic snapshots of |*ψ*|^2^ when we are in this regime. Conversely, when the Josephson-like vortex is involved [points (3) and (4)], the vortex core extension becomes more and more undefined (increases) and the corpuscular nature of this vortex surfing on a river of further depressed superconductivity is more and more lost.

At very low magnetic field only one vortex excitation is involved both in the flux flow branch (Abrikosov-Josephson) and phase slip branch (Josephson-like). This allows us to estimates the velocities involved in our weak link directly from experimental *V*(*I*) curves in the very low field regime. In physical units we can estimate the mean velocity from the measured voltage and width of the weak link as *v* = *WV*/Φ_0_. From curves at 4.25 K reported in Fig. 2(b) we can assume that, e.g., the curve at *B* = 1.26 mT is accounted for a single vortex excitation. From this we see that typical voltage in the flux branch is *V*_*AJ*_ ~ 10 *μ*V, giving a velocity *v*_*AJ*_ ~ 5 × 10^3 ^m/s. This velocity is of the order of magnitude we reported for ordinary Abrikosov vortex motion in mesoscopic strips^49^, though here it is sensibly larger, being a weak link involved. Conversely, from same curve, we read a typical voltage in the phase slip line branch *V*_*Jl*_ ~ 120 *μ*V resulting in a velocity for the Josephson-like vortex *v*_*Jl*_ ~ 6 × 10^4^ m/s, that is an order of magnitude larger than the Abrikosov-Josephson vortex, though lower than typical velocity of fastest known vortex excitation, i.e., the Josephson vortex in tunnel junctions^26^, where typically *v*_*J*_ ~ 3 × 10^6^ m/s is observed.

**Figure 6:**
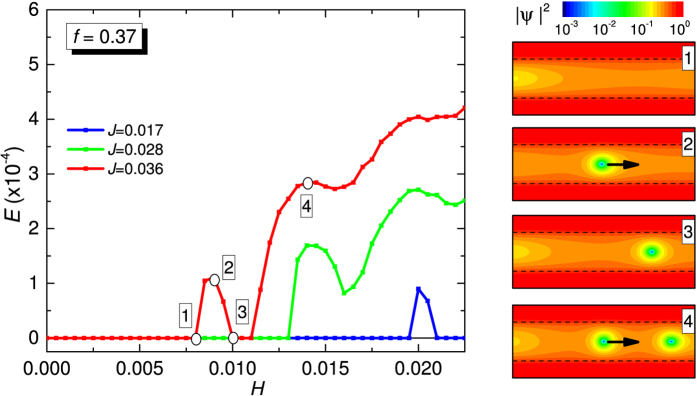
Magnetoresistance oscillations induced by strongly confined vortex matter. Time-averaged electric field *E* as a function of magnetic field *H* for different values of applied current density *J*. The numbered panels are snapshots of |*ψ*|^2^ at the field and current values indicated in the *E*(*H*) curves. Arrows in panels (2) and (4) indicate direction of vortex matter motion.

We conclude with a short analysis of magneto-resistance oscillations observed in the experiments. The calculated *E*(*H*) curves at different values of applied current density *J* are reported in Fig. 6. These curves should be compared with experimental data shown in Fig. 2(c). As in the experiments, the full reentrance of superconductivity (red curve) corresponds to 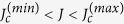. The numbered panels are snapshots of |*ψ*|^2^ at the field and current values indicated in the *E*(*H*) curves. Arrows in panels (2) and (4) indicate direction of vortex matter motion. For red curve the current is fixed at *J* = 0.036. At *H* = 0 there are no vortices in the weak link. By increasing the field, a current-assisted Abrikosov-Josephson vortex nucleation at left edge is achieved at a critical value [point (1)] and vortex moves under the influence of Lorentz force producing dissipation (*E* ≠* *0). By increasing the magnetic field, the vortex is first accelerated [point (2)] and then stopped [point (3)] near the right edge at some critical value of magnetic field, so that the fully superconducting state (*E* = 0) is restored. The vortex stays at rest, due to confining potential of magnetic field, until a further increase of magnetic field produces a new vortex nucleation at left edge. Due to the repulsive inter-vortex interaction, the new vortex expels the static vortex and dissipation is again established [point (4)].

The shape of *E*(*H*) oscillations, stemming from *J*_*c*_(*H*) modulations, strongly reminds the one reported in a recent numerical work^41^ by Berdiyorov *et al*. on weak links. In that case a rather short and narrow weak link was addressed, but also if our real weak link is slightly longer and wider, here we confirms that involved dynamics is practically the same as the one described by Berdiyorov *et al*., and it is typical^3^,^4^,^41^ of strong confinement of a very small number of vortices (here only one or two). In this sense, the data in Fig. 2(c) are the experimental demonstration of the effect predicted by Berdiyorov *et al*. specifically for a weak link, and add further experimental evidence^5^,^6^,^7^,^8^ for the peculiarities^3^,^4^,^41^ of magneto-transport in strongly confined vortex matter systems.

## Methods

Both thin Nb and Py films were deposited by rf magnetron sputtering in a high vacuum system with a base pressure of 2 × 10^−7^ Torr at room temperature. The geometry of both films was defined by lift-off through a resist mask made by electron beam lithography. To define the weak links, first a 30-nm thick Nb film is deposited onto a Si/SiO_2_ substrate and patterned with a multi contact geometry. Then, after a light sputter etch of the Nb layer (3 min at 400 V, enough to eliminate the thin thermal oxide grown after exposition to air) 30-nm thick Py stripes are deposited to locally cross the Nb strip. A micrograph of final sample layout is shown in the inset of Fig. 1(a). The horizontal Py stripes are all 10 *μ*m long and their widths are 0.2 *μ*m, 0.4 *μ*m, 0.6 *μ*m, and 0.8 *μ*m. The vertical section of Nb strip is 1 *μ*m wide, so that all weak links are 1 *μ*m wide, and the lateral fingers, used for voltage contacts, are 5 *μ*m apart. The black lettering in the micrograph shows the current and voltage electrodes used for four-point electrical measurements.

We notice that due to the presence of a Nb/Py bilayer with variable length *L*_*SF*_ = *xL*_*V*_ between the fixed voltage probes distance *L*_*V*_ = 5 *μ*m the fully normal state resistance is not expected to be exactly the same for all samples, as it is in fact observed in Fig. 1(a). The Nb banks have variable length *L*_*S*_ = (1 − *x*)*L*_*V*_ and their resistance is *R*_*S*_(*x*) = (1 − *x*)*R*_*N*_(0) with 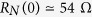 the resistance of reference sample. Resistivities of our Nb and Py are very similar, the thickness is the same, and a good electrical contact between Py and Nb layer was intentionally made. Thus, the resistance of the weak link portion is approximately *R*_*SF*_(*x*) = (*x*/2)*R*_*N*_(0). At 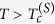 both banks and SF regions are normal so that we measure a resistance *R*_*N*_(*x*) = (1 − *x*/2)*R*_*N*_(0) decreasing as the weak link region length is increased, in agreement with results shown in Fig. 1(a). Conversely, in the temperature range 
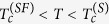
 only the SF region is normal and, apart for corrections due to lateral proximity effect, a resistance *R*_*SF*_(*x*) ~ (*x*/2)*R*_*N*_(0) increasing with length of the weak link should be observed, in qualitative agreement with results of Fig. 1(a). Particularly, for the longest weak link is 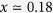 and we measure in the plateau region a resistance of the SF bilayer 4 Ω < *R*_*SF*_ < 6 Ω. Such a figure is consistent with 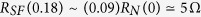, the resistance expected when the proximity effect in the vertical direction (depletion of superconductivity) affects the entire thickness and surface of the Nb below the Py.

To minimize thermal effects, measurements were made in a liquid Helium dewar and the Si/SiO_2_ substrate was glued with silver paint to a massive copper sample holder. The voltage-current curves were acquired in a reasonably short time (typically 50 s) and permanence in dissipative states that could generate thermal instability or hot spots was carefully minimized. Electrical interference was minimized filtering all cables with RC filters and external magnetic field interference was minimized by means of a high permeability cryoperm shield. Very low noise voltage-current curves were recorded using a current source/nanovoltmeter combo, and a similar electronics already used for voltage-spectral density characterizations^55^. Stable low magnetic field was applied by means of a copper solenoid held in Helium bath biased by a precision current source.

From the *I*_*c*_(*B*), recorded at *T* = 4.25 K, shown in Fig. 2(a) we can gain information on the electrical width^26^ of the weak link with respect to the Josephson penetration length *λ*_*j*_^26^ often used for weak links. The first lobe extrapolates to *I*_*c*_ = 0 at a critical field^26^ 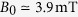. Assuming the presence of a Josephson coupling, we can approximately estimate the normalized electrical width 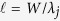 of our weak link using same formula^26^,^40^ for *λ*_*j*_ used in tunnel junctions. From the measured critical current density *J*_*c*_ at zero field and the magnetic penetration^26^ *d* = 2*λ* + *L*, where *L* = 0.2 μm is the length of the weak link and *λ* is the temperature-dependent London penetration depth of the Nb thin film (see below), we estimate *λ*_*j *_≈ 0.25 *μ*m, resulting in a normalized width 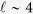. On the basis of this, we expect that a weak magnetic field can induce a small number (up to to a maximum of four) of vortex excitations in the weak link. In numerical simulations shown in Fig. 4(a) we see that at the entry field (critical field) two almost isotropic (static) vortices are present in the weak link, meaning that the effective magnetic flux Φ(*B*) = *BA*_*e*_, should be practically equal (in physical units) to 2Φ_0_ at *B* = *B*_0_. From this we can estimate an effective magnetic area of the weak link as 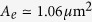, that is found to be consistent with the one expected by the relation^26^ 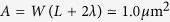 using real physical dimensions of the weak link and noticing that at 4.25 K for our Nb is 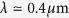. As the resistance based check, this magnetic check confirms that the effective area of the realized weak link is the one geometrically designed, and no other mechanisms beside (full) proximity effect are relevant.

In model equations (1), and (2) the coordinates are in units of coherence length at working temperature 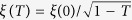, with temperature *T* in units of critical temperature of banks 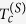; time is measured in units of the relaxation time *τ*(*T*) = *τ*(0)/(1 − *T*); the order parameter is in units of 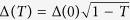; the vector potential is measured in units Φ_0_/2*πξ*(*T*); the electrostatic potential is in units of 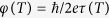. In these units the magnetic field is scaled with *H*_*c*2_(*T*) = Φ_0_/2*πξ*(*T*)^2^ and the current density with *j*(*T*) = *c*Φ_0_/8*π*^2^*λ*(*T*)^2^*ξ*(*T*), with 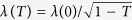. In our numerical integration of equations (1) and (2) we make use of the “bridge” boundary condition^4^,^56^,^57^ in the *y*-direction: in a region of length 2.5 *ξ* at the ends of the banks at *y* = ± *L*_*B*_/2 [see insets of Fig. 3(a)] the TDGL equations are reduced to (∂_*t*_ + *iϕ*)*ψ* = 0, ∇^2^*ϕ* = 0 with boundary conditions ∂_*y*_*ψ* = 0 and ∂_*y*_*ϕ* = −*J*, where *J* is the uniformly injected bias current density. This mimics a situation where in the region of interest the current is injected from superconducting contacts, as it is in the experiment [see insets of Fig. 1]. In the *x*-direction, an insulator-superconductor boundary condition (*i*∂_*x*_ + *A*_*x*_)*ψ* = 0 is used for the order parameter and the Neumann boundary condition ∂_*x*_*ϕ* = 0 is applied for the electrostatic potential. The initial conditions are |*ψ*| = 1 and *ϕ* = 0. We apply a finite-difference representation for the order parameter *ψ* and the electrostatic potential *ϕ* on a uniform Cartesian space grid and use the link variable approach^58^. We employ a Dormand-Prince embedded method for ordinary differential equations (an embedded Runge-Kutta integrator of order 8 with stepsize control) to find *ψ*. The electrostatic potential *ϕ* is obtained by the Fourier transform method. When dc quantities are involved, the behaviour of the system is studied on a large time scale when time-averaged values no longer depend on time.

From the slope close to *T*_*c*_ of the upper critical field *μ*_0_*H*_*c*2_(*T*) of the reference strip we estimate^59^ for our sputtered Nb film a coherence length 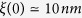. From resistivity and critical temperature we estimate^51^ a London penetration depth 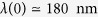, resulting in a Ginzburg-Landau parameter 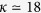. So, our thin Nb strip is definitively a type-II superconductor. Moreover, a Pearl length Λ_* *_≈ 2.1 *μ*m is estimated for our 30-nm thick Nb at *T* = 0 that increases at Λ_* *_≈ 4.2 *μ*m at reduced temperature *T* ~ 0.8. So, noticing that the strip width is *W* = 1 *μ*m and that experiments were performed at *T* ≥ 0.8 the conditions for mesoscopic regime are reasonably satisfied for our strips.

## Additional Information

**How to cite this article**: Carapella, G. *et al*. Current driven transition from Abrikosov-Josephson to Josephson-like vortex in mesoscopic lateral S/S’/S superconducting weak links. *Sci. Rep.* **6**, 35694; doi: 10.1038/srep35694 (2016).
